# Dielectric Spectroscopy of Hybrid Magnetoactive Elastomers

**DOI:** 10.3390/polym13122002

**Published:** 2021-06-18

**Authors:** Vitaliy G. Shevchenko, Gennady V. Stepanov, Elena Yu. Kramarenko

**Affiliations:** 1 Enikilopov Institute of Synthetic Polymeric Materials of Russian Academy of Sciences (ISPM RAS), 117393 Moscow, Russia; shev@ispm.ru; 2 State Scientific Center of the Russian Federation, Institute of Chemistry and Technology of Organoelement Compounds, 111123 Moscow, Russia; gstepanov@mail.ru; 3 Faculty of Physics, Lomonosov Moscow State University, 119991 Moscow, Russia; 4 A. N. Nesmeyanov Institute of Organoelement Compounds RAS, 119991 Moscow, Russia

**Keywords:** magnetoactive elastomers, magnetic elastomers, magnetically hard particles, magnetodielectric effect, dielectric constant

## Abstract

Dielectric properties of two series of magnetoactive elastomers (MAEs) based on a soft silicone matrix containing 35 vol% of magnetic particles were studied experimentally in a wide temperature range. In the first series, a hybrid filler representing a mixture of magnetically hard NdFeB particles of irregular shape and an average size of 50 μm and magnetically soft carbonyl iron (CI) of 4.5 μm in diameter was used for MAE fabrication. MAEs of the second series contained only NdFeB particles. The presence of magnetically hard NdFeB filler made it possible to passively control MAE dielectric response by magnetizing the samples. It was shown that although the hopping mechanism of MAEs conductivity did not change upon magnetization, a significant component of DC conductivity appeared in the magnetized MAEs presumably due to denser clustering of interacting particles resulting in decreasing interparticle distances. The transition from a non-conducting to a conducting state was more pronounced for hybrid MAEs containing both NdFeB and Fe particles with a tenfold size mismatch. Hybrid MAEs also demonstrated a considerable increase in the real part of the complex relative permittivity upon magnetization and its asymmetric behavior in external magnetic fields of various directions. The effects of magnetic filler composition and magnetization field on the dielectric properties of MAEs are important for practical applications of MAEs as elements with a tunable dielectric response.

## 1. Introduction

The development of new smart materials that allow controlling their properties using external stimuli is one of the complex interdisciplinary tasks facing various fields of modern science, such as materials science, chemical engineering, chemical physics, and others. Nowadays, smart materials are widely used in science and technology, and there is an ever-growing need for new materials. Introducing several functionalities into one material capable of performing multiple functions is especially difficult. One of the ways to design multifunctional materials is to combine species of different chemical nature, which possess different physical properties, in one composite to create a synergetic combination of these properties within a new complex material. A striking example is so-called magnetoactive elastomers (MAEs), consisting of a soft polymer matrix with embedded ferromagnetic micro- and/or nanoparticles [1,2]. Combining the elasticity of the polymer with the magnetism of the filler has proven to be very successful in creating a high magnetic response of these materials [1,2,3,4,5,6,7,8,9]. The presence of magnetic particles introduces magnetic sensitivity, while the elastomeric nature of the dispersing medium allows external control of various physical properties of the resulting composites via the magnetomechanical coupling that occurs when a magnetic field is applied and manifests itself by the restructuring of magnetic particles in the soft elastomer matrix. This restructuring is driven by magnetic interactions favoring alignment of magnetic particle aggregates along the field lines that are limited by the elasticity of the matrix. One of the most studied effects raised in these materials is the so-called magnetorheological effect, i.e., a considerable change of mechanical and viscoelastic properties of MAEs in magnetic fields [10,11,12]. However, some other phenomena have also attracted much attention. Among them are magnetodeformational effect [13,14,15,16,17,18], i.e., significant deformations of MAE samples in homogeneous and gradient or alternating magnetic fields, magnetodielectric effect as well as magnetoresistance of MAEs [19,20,21,22,23], i.e., a tremendous increase in both relative permittivity and conductivity of samples in homogeneous magnetic fields. Furthermore, MAEs demonstrate magnetically controlled radio-absorbing properties [24,25,26] that are promising for the design of magneto-controllable radio-shielding or polarizing covers on their basis. Advances of MAE development have been reviewed in several recent publications [3,4,5,6,7,8], while possible practical applications of these exciting materials have been described in detail, for example, in Ref. [27]. Recently, magneto-polymer composites have been used for the design of soft robotic systems [28,29].

Most studies in the field of MAEs have been carried out for materials based on magnetically soft (MS) particles, in particular, carbonyl iron (CI) [3]. MS filler allows active control of its magnetization state and, thus, MAEs properties by external magnetic fields. Increasing magnetic field leads to an enhancement of magnetic interactions shifting the balance between magnetic and elastic forces acting on filler particles and favoring their displacements from initial equilibrium positions with the formation of tighter aggregates. After the magnetic field is switched off, demagnetized MS particles return to their initial positions due to restoring elastic forces, and an MS MAE takes its original state. Recently much interest has also been paid to magnetic composites containing magnetically hard (MH) filler particles [30,31,32,33,34,35]. Contrary to magnetically soft materials, magnetically hard ones keep their magnetization state after the removal of the magnetic field. As a result, MH filler enables a passive control of the physical properties of the resulting MAEs, which can be considerably changed once upon magnetization [30,31]. In particular, the elastic modulus of magnetically hard MAEs increases with an increase in the magnetization field [32,33]. A combination of active and passive control can be achieved in so-called hybrid materials containing a mixture of magnetically soft and hard fillers [34,36,37,38,39]. The passive control of MH MAEs is attained upon initial sample magnetization, while the active control with a further variation of an external magnetic field is promoted by strong interactions of MH and MS filler particles. Hybrid MAEs are quite new materials, and up to now, the main focus was on their magnetic properties as well as mechanical and viscoelastic response to external magnetic fields [34,36,37,38,39].

In this paper, we focus on the dielectric properties of hybrid MAEs and explore the possibility of their control via material magnetization after its preparation. To our knowledge, this is the first attempt of dielectric spectroscopy study of these composites. Upon magnetization, the average distance between the conducting particles within magnetically stabilized aggregates is expected to decrease, affecting the dielectric/electric properties of these materials. We demonstrate that, indeed, the magnetization of hybrid MAEs can serve as an effective tool for substantially tuning their electric and dielectric response. It should be mentioned that the magnetodielectric effect (MDE) and magnetoconductivity of MS and MH MAEs in an applied magnetic field have been previously studied in a number of publications [19,20,21,22,23,40,41,42,43,44,45,46,47,48,49,50]. Within the general approach, the value of MAE permittivity can be obtained from the measurements of the capacity of a capacitor filled with an MAE [22,23,40,41,42]. A strong increase in the capacitance was observed when the magnetic field was applied perpendicular to the capacitor plates [40,41]. At a fixed distance between the capacitor plates during measurements, the capacitance change characterizes a change in the effective permittivity of the material caused by filler re-arrangements within the polymer matrix [22,23,41]. The effect of the type of magnetic filler, as well as the size and concentration of magnetic particles within composites, was studied in [22]. The largest MDE of the order of 1000% in the magnetic field of 0.57 T was reported for MAEs based on silicone matrix containing 75wt% of CI [41]. Simple theoretical models [22,23,48] as well as computer simulations [49] confirm that MDE originates from the restructuring of the magnetic filler within a soft elastomer matrix. The recently developed theoretical approach [50], based on the effective medium approximation with the account of field-dependent restructuring of the filler, described the observed magnetodielectric phenomena in MAEs rather well.

A significant increase in the electrical conductivity of polymers filled with CI can be achieved in anisotropic MAEs synthesized in an external magnetic field [46,47] because produced anisotropy significantly reduces the percolation threshold. In this paper, we demonstrate that MH filler particles within hybrid MAEs give us an opportunity to induce an anisotropy and, thus, to control MDE and conductivity by application of magnetic field after material fabrication. This is promising for the design of magnetic field sensors or controlled shielding materials on the basis of hybrid MAEs.

In the next section, the composition, the method of synthesis and study of MAEs samples are introduced. In Section 3, we first discuss the dielectric characteristics of hybrid MAEs based on the mixture of NdFeB and Fe particles and make a comprehensive analysis of the effect of sample magnetization on its dielectric properties. Then, a similar comparative analysis of the dielectric response of MAEs containing only magnetically hard NdFeB filler is presented. The main results are summarized in the Conclusion section.

## 2. Materials and Methods

Magnetoactive elastomers were synthesized on the basis of the silicone compound SIEL [51], which has many advantages for producing smart MAEs due to the softness of the resulting composites, wide operating temperature range and biocompatibility [32]. We used two types of commercially available magnetic fillers which were magnetically soft carbonyl iron (spherical particles with the average diameter of 4.5 μm (grade R-20, OOO SINTES-PKG, Russia) and magnetically hard alloy of NdFeB (Powder-YMM-Q(8-3) Yuhong Magnetic Materials Co., Ltd., Jiangmen, China). Particle characterization of these fillers has previously been performed in Refs. [22,33]. MH NdFeB is irregularly shaped particles with a wide size distribution from 1 to 100 μm (see Figure S1)). To enhance compatibility with the silicone polymer matrix, magnetic particles were modified by a mixture of hydrophobic agent ((CH_3_)_3_Si–O–[CH_3_Si(H)–O–]_n_–Si(CH_3_)_3_) with silicone oil in hexane. The mixture was treated by ultrasound for 2 min and dried (see [32], for details). After drying, a hydrophobic film remains on the particle surface.

The fabrication of MAEs has previously been described in detail in [8,11,15]. Shortly, the silicon compound SIEL comprising of a low-molecular vinyl-containing rubber (component A) and a hydride-containing cross-linking agent (component B) in the proportion A:B = 9:1 was mixed with pre-treated magnetic particles and carefully homogenized using a roll powder dispenser. The catalyst was introduced into this pre-composition that was further degassed in vacuum at a pressure of about 1–2 mmHg. Then, the mixture was poured into a mold, and the polymerization was carried out at 150 °C. An external magnetic field of 80mT was applied vertically during polymerization to prevent particle sedimentation. The application of a weak magnetic field is expected to cause also a rotation and a slight alignment of the particles along the field lines [25,32,52,53].

Following the above procedure, two series of ribbon-like MAE samples were obtained. The amount of the magnetic filler in both series was equal to 35 vol%; however, the composition of the filler was different. For the first type of MAE samples, a mixture of NdFeB and Fe particles in the mass proportion 80:20 was used (we call them hybrid composites). The other series was based on the pure magnetically hard NdFeB filler.

For dielectric measurements, the disk-shaped samples with a diameter of 20 mm and a height of 1.1 mm were cut from the ribbons. To elucidate the effect of the magnetizing field on the dielectric properties of the MAEs, some samples were magnetized in the magnetic field of 1.5 T for 30 min. The magnetizing field was directed perpendicular to the sample surface. The sample characteristics are summarized in Table 1.

The real and imaginary parts of the relative permittivity and the loss factor were measured for all samples using an impedance analyzer Novocontrol Alpha-A (Installation Concept 40) and the dielectric cell ZGS Alpha Active Sample Cell with gold disk electrodes with a diameter of 20 mm. Measurements were performed in the frequency range of 10^−1^–10^6^ Hz. Electrodes of 20 mm in diameter made from 10 mm thick Al foil were pasted on the two opposite surfaces of each disk sample. The foil electrodes were glued to the sample surface with silver-containing conductive adhesive D-550 produced by Dotite, Japan. Measurements were carried out in the temperature range from −30 °C to +50 °C, where the most significant changes in electrical properties took place. It should be noted that during the spectroscopy measurement of magnetized samples, the applied electric field was oriented in the same direction as the magnetization field.

**Figure 1:**
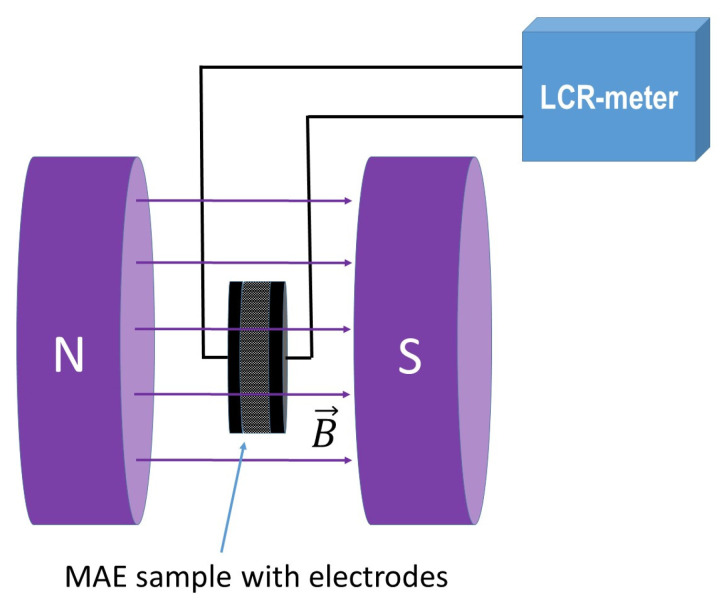
A schematic of a setup for studying the effect of a magnetic field on the dielectric permittivity of MAEs.

The dependence of the MAE sample capacity on the magnetic field was measured at room temperature using an Agilent E4980A LCR meter, placing the samples between the poles of an electromagnet included in the LakeShore 7407 Hall effect system (Lake Shore Cryotronics Inc., Westerville, OH, USA). The magnetic field was monitored with a vibrating magnetometer with an accuracy of ±0.01 mT. The direction of the magnetic field was perpendicular to the sample surface (see Figure 1).

## 3. Results and Discussions

### 3.1. Hybrid MAE Composites Based on NdFeB and Fe Particles

**Figure 2:**
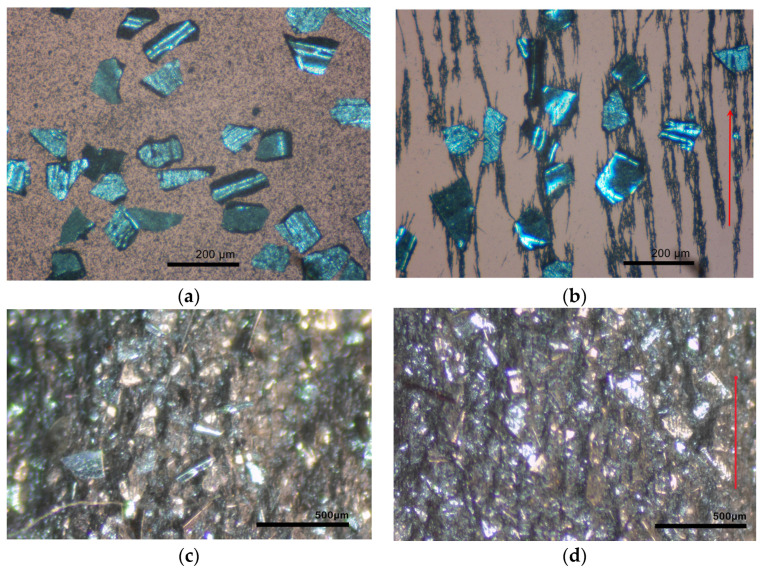
Optical micrographs of a mixture of NdFeB and Fe particles in silicone oil (**a**) in the absence of any magnetic field and (**b**) after the magnetization in the magnetic field of 1T (the red arrow shows the direction of the magnetizing field). Optical micrographs of a section of MAE–Fe sample (**c**) after preparation and (**d**) after magnetization in the magnetic field of 1.5 T.

In this section, we discuss the dielectric properties of the hybrid MAEs based on the mixture of NdFeB and Fe particles and elucidate the effect of the magnetizing field on the dielectric constant and conductivity of these composites. One can expect that the structure of the magnetic filler within the polymer matrix would change in the course of magnetization due to the clustering of magnetic particles and their alignment along the field direction, as has been shown in [34]. This process is illustrated in Figure 2, where we present optical micrographs of a thin layer of a mixture of large-size-fraction NdFeB and Fe particles in silicone oil, which was used for MAE–Fe fabrication, in the absence of magnetic field (Figure 2a) and after application of the magnetic field of 1T in the direction parallel to the layer surface (Figure 2b). One can see that smaller CI particles form needle-like aggregates connecting larger NdFeB particles. Magnetized MH NdFeB keeps its magnetization state after the removal of the external field, and CI filler remains structured, interacting with the field of NdFeB forming clusters. A similar restructuring is expected for MAE–Fe composites during magnetization, although its degree is limited by the elasticity of the polymer matrix, which prevents large displacement of particles from their initial positions. Figure 2c,d demonstrate photographs of a section of the MAE–Fe sample after preparation and immediately after magnetization, respectively. At high degrees of filling, such as those used in this work, magnetic structures in 3D are difficult to visualize; however, some alignment of the particles upon magnetization can be seen by comparing Figure 2c,d. It should be noted that the structuring of MH particles within silicone matrices was reported in [52,53], where X-ray microtomography was used to analyze the particle movement induced by magnetic fields.

**Figure 3:**
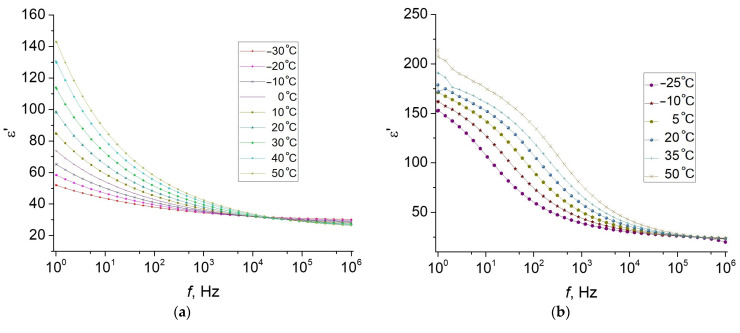

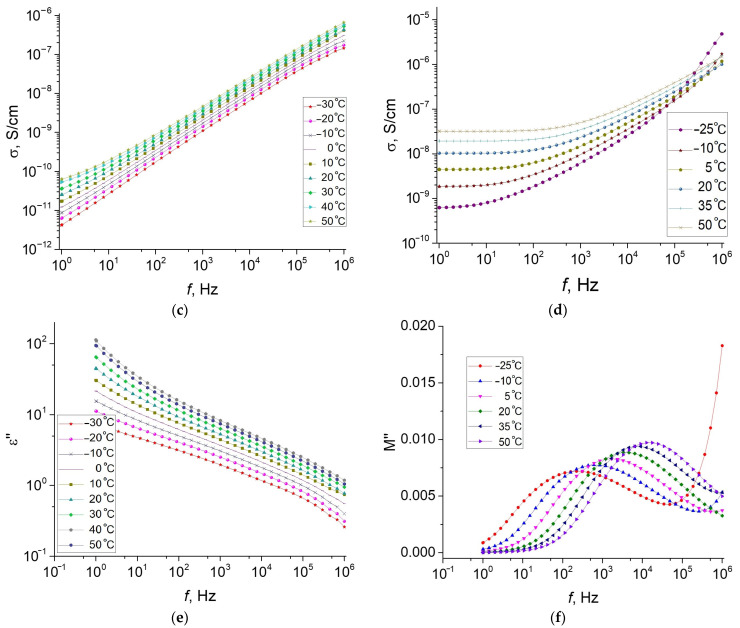
Frequency dependence of (**a**,**b**) the dielectric constant and (**c**,**d**) conductivity as well as (**e**) the dielectric losses and (**f**) the modulus, measured at various temperatures for (**a**,**c**,**e**) the original MAE–Fe and (**b**,**d**,**f**) magnetized MAE–Fe–m samples.

Figure 3 shows the dielectric characteristics of the hybrid MAEs (the samples MAE–Fe and MAE–Fe–m) as functions of frequency at various temperatures. As can be seen, all the dependences differ significantly for the original (Figure 3a,c,e) and magnetized (Figure 3b,d,f) composites. The magnetized sample MAE–Fe–m is characterized by much larger values of the dielectric permittivity (Figure 3b) than the non-magnetized one, and the frequency dependences of the conductivity demonstrate the presence of a DC conductivity $\sigma_{dc}$ in the magnetized MAE–Fe–m, which is reflected by the appearance of characteristic plateaus. In this case, the imaginary dielectric permittivity is inversely proportional to frequency, exhibiting common behavior. Due to a rather high conductivity in the magnetized composite, the dielectric losses are high; therefore, in Figure 3f, we plot the frequency dependences of the imaginary part of the electric modulus, $M^{\prime \prime }$, for the magnetized sample. This presentation makes it possible to eliminate the effects of electrode polarization and conductivity. The electric modulus is defined as the inverse of the complex dielectric constant:

(1) $$M^{*}=\frac{1}{\varepsilon^{*}}=\frac{1}{\varepsilon^{\prime }-i\varepsilon^{\prime \prime }}=M^{\prime }+iM^{\prime \prime }$$

where $\varepsilon^{\prime },\;\varepsilon^{\prime \prime },\;M^{\prime },\;M^{\prime \prime }$ are the real and imaginary parts of the dielectric constant and electric modulus, respectively.

AC conductivity ($\sigma_{ac}$) includes all the effects of the dissipation of the electromagnetic field energy, including ohmic conductivity when carriers move along isolated centers or clusters, as well as dielectric losses [54]. In solids containing phases with different conductivities, the electrical conductivity increases with increasing frequency [55,56]. At low frequencies, carriers are forced to move longer distances over a half-period of the electric field, and their transfer is difficult because conductive clusters are isolated from each other, whereas at high frequencies, local carrier movement occurs within conducting clusters [57,58].

The dispersion of the conductivity $\sigma_{ac}$ is characteristic of all heterogeneous and disordered solids [56,59,60]. Obviously, MAEs also belong to such systems since they are not metals and do not have the correct crystal lattice [59]. For a reason not yet sufficiently understood, for a very wide range of materials, the frequency dependence of $\sigma_{ac}$ has the same form, which is common to all disordered solids [56,61,62]. This dependence at a constant temperature is the following [62,63]:

(2) $$\sigma_{ac}\left(\omega \right)=\sigma_{dc}+A{\left(\omega \right)}^{s}$$

where $\sigma_{dc}$ is the limiting value of $\sigma_{ac}$ at ω→0; *A* and *s* are the parameters that depend on filler concentration and temperature [59,64]. This expression and the model from which it is derived are often called the universal dynamic response because of their applicability to a large number of systems [62].

**Figure 4:**
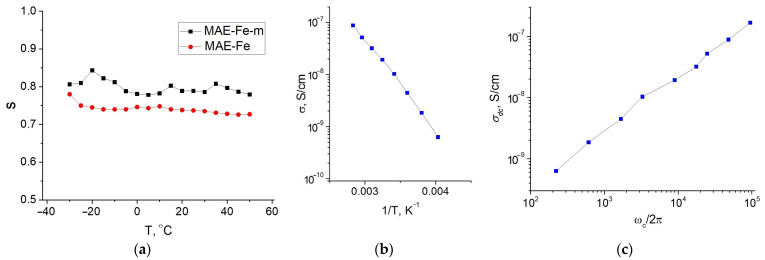
(**a**) Temperature dependence of the parameter *s* for MAE–Fe and MAE–Fe–m samples; (**b**) Arrhenius-type dependence of the conductivity of MAE–Fe–m; (**c**) log-log plot of $\sigma_{dc}$ on the critical frequency $\omega_{c}$ for MAE–Fe–m.

Figure 4a shows the temperature dependence of the parameter *s* in the frequency dependence of $\sigma_{ac}$ (Equation (1)) obtained for the original MAE–Fe and the magnetized MAE–Fe–m samples. As can be seen, for both samples, the parameter *s* weakly depends on temperature; however, its value is slightly higher for the magnetized sample. The effective activation energy determined from the Arrhenius-type dependence of the conductivity (see Figure 4b) is equal to 0.55 eV. Thus, the obtained results indicate that the conductivity has a hopping character. In this case, the non-magnetized composite is in a dielectric regime of conductivity, while the magnetized one is characterized by a significant component of the DC conductivity $\sigma_{dc}$. It means that during the magnetization of MAEs, a transition from a non-conducting to a conducting state takes place. In other words, during magnetization, the composite experiences a transition from a regime below the percolation threshold to that above the threshold, apparently due to a contraction of particle clusters, which results in a considerable decrease in the average distance between the filler particles.

A similar distinction in dielectric behavior has been reported for isotropic and anisotropic MAEs based on iron particles [46]. The later ones were synthesized in an external magnetic field, and the chain-like structure oriented along the field line aggregates was introduced in the course of polymerization. The isotropic MAEs demonstrated no conductance, while all their anisotropic analogues showed the clear presence of DC conductivity contribution in the frequency dependence of AC conductivity. This distinction in behavior was explained by the formation of percolation clusters in anisotropic samples at rather low concentrations of filler particles when isotropic counterparts of the same concentrations were far from the percolation threshold [46].

The maxima on the frequency dependences of the imaginary part of the electrical modulus (Figure 3f) correspond to the critical frequency $\omega_{c}$ at which the dispersion on the frequency dependences of conductivity appears. According to the work of Barton/Nakajima/Namikawa [56,65,66,67], $\sigma_{ac}\sim \omega_{c}$ for the hopping mechanism of conductivity (the so-called BNN relation). As can be seen from Figure 4c, this linear relationship is valid for the magnetized composite, indicating that the conduction mechanism does not change when passing from a dielectric to a conducting mode upon sample magnetization. A sharp increase in $M^{\prime \prime }$ at high frequencies at −25 °C is probably related to some transition in the polymer matrix, affecting the transport of charge carriers [68]. This effect requires additional research, which is beyond the scope of this article.

**Figure 5:**
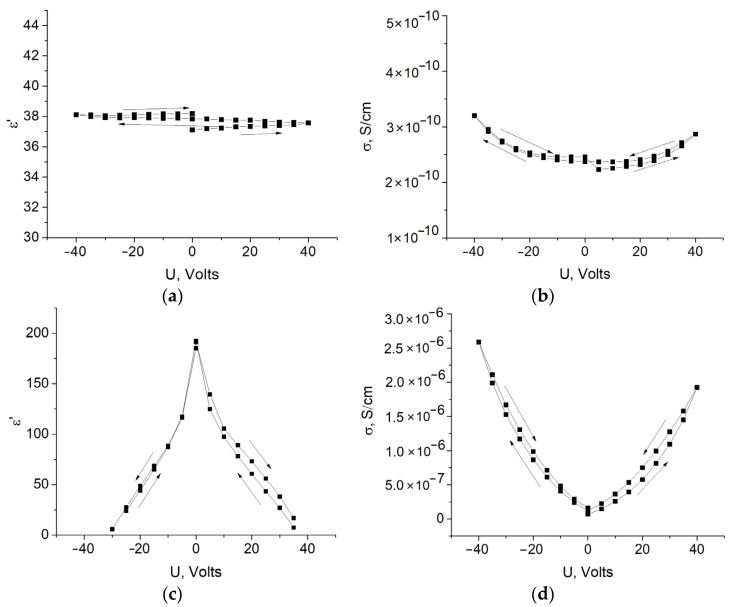
Dielectric constant (**a**,**c**) and conductivity (**b**,**d**) versus DC bias voltage for MAE–Fe (**a**,**b**) and MAE–Fe–m (**c**,**d**). *f* = 100 Hz.

The hybrid composites were found to exhibit a dependence of the electrical properties on the presence of a constant bias voltage. The dielectric properties were measured by applying a dc bias voltage in the sequence 0→40→0→−40→0 B. The results are shown in Figure 5. For the non-magnetized composite, the effect is small, while after magnetization, the presence of a constant bias voltage has a very strong effect on the measured values. The high values of the dielectric constant are apparently associated with the polarization of the interface when charges are accumulated in the polymer–particle interphase layer [69]. The application of dc voltage displaces charges away from this layer, reducing their concentration and, respectively, decreasing MWS (Maxwell-Wagner-Sillars) permittivity.

### 3.2. FeNdB Composites

In this section, the dielectric properties of MAEs containing only MH NdFeB particles are studied and compared with those of the hybrid composites.

**Figure 6:**
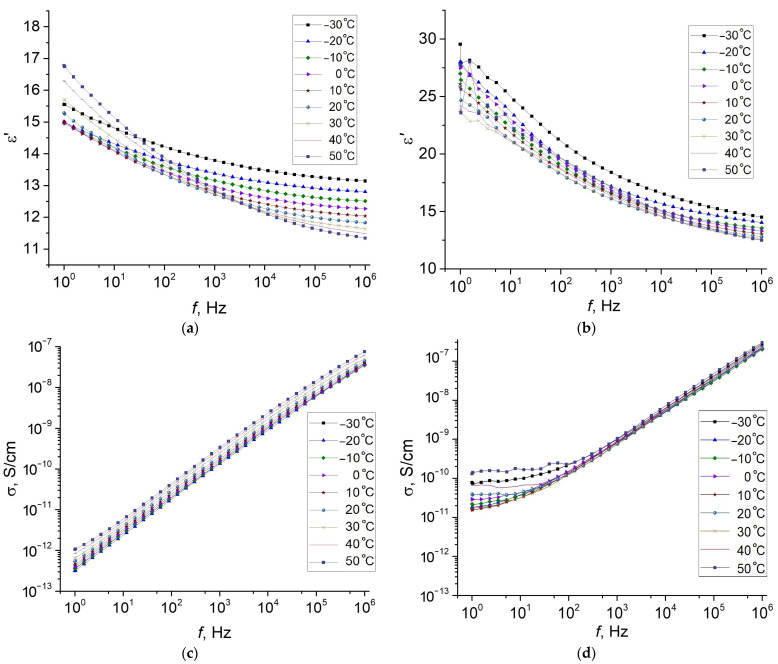
Frequency dependence of the dielectric permittivity (**a**,**c**) and conductivity (**b**,**d**) of MAE–0 (**a**,**b**) and MAE–0–m (**c**,**d**) at various temperatures.

Figure 6 shows the frequency dependences of the dielectric constant and conductivity at different temperatures for the original MAE–0 and the magnetized sample MAE–0–m. The temperature dependences at different frequencies can be found in SI, Figure S3. Compared to the hybrid composites, the dielectric constant is noticeably lower, although its value is much higher for the magnetized sample MAE–0–m in comparison with the original MAE–0. As in the case of hybrid composites, the magnetized sample MAE–0–m is characterized by a dispersion of conductivity (Figure 6c), but it is less pronounced.

It can be assumed that the temperature dependences of the dielectric constant and conductivity for composites based on NdFeB are mainly determined by the thermal expansion of the matrix polymer, which leads to an increase in the distance between conducting particles and, as a consequence, a decrease in the mutual capacity of particles and a corresponding decrease in the dielectric constant of the composite. The dependences of $\varepsilon^{\prime }$ on temperature (the corresponding dependence at the frequency of 10^6^ Hz is shown in the inset of Figure S3) follow with great accuracy the equation $\varepsilon^{\prime }=A-B\log\left(T+C\right)$, where *T* is the temperature, *A*, *B*, *C* are some parameters.

Consider the following model of the composite. The particles are evenly located at the nodes of the cubic lattice and are spherical for simplicity. The behavior of the dielectric characteristics in a first approximation is determined by the properties of a capacitor formed by two adjacent spherical particles with metallic conductivity. The average distance *h* between the surfaces of spherical particles of diameter *d* randomly distributed in the volume of polymer can be determined from the following equation [70]:

(3) $$h=d\left[{\left(\frac{\pi }{6\varphi_{f}}\right)}^{1/3}-1\right]$$

where $\varphi_{f}$ is the volume fraction of the filler. The filler content in MAEs is equal to 35 vol%. Provided that particles with a diameter of 100 μm are uniformly distributed, the average distance between the surfaces of the particles is 20 μm.

Since the distance between the particle centers, *r*, approaches the particle diameter, *d*, we can consider the solution of Maxwell’s equations for the mutual capacity of two spheres in the near-field approximation [71]. For further analysis, it is convenient to introduce the parameter ξ=(r−d)/d, which is the relative distance between the surfaces of the spheres. Then the mutual capacity $C_{ab}$ in the approximation of the first terms of the series can be written in the form

(4) $$c_{ab}\equiv C_{ab}/C_{aa}=-\frac{1}{4}\ln\xi +\frac{\gamma }{2}-\frac{1}{4}\ln2+O\left(\xi \ln\xi \right)$$

where, γ = 0.577216 is Euler’s constant, and $C_{aa}$ is the intrinsic capacitance of each sphere.

Assuming the thermal expansion of the polymer matrix and neglecting the thermal expansion of the filler, we can write that $\xi =\xi_{0}+\alpha T$, where α is the coefficient of thermal expansion, *T* is the temperature. Substituting it into Equation (2), we obtain an expression of the form $c_{ab}=a_{1}-a_{2}\ln\left(T+a_{3}\right)$. Because the dielectric constant is related to $c_{ab}$, the temperature dependence of $\varepsilon^{\prime }$ should be the same. This is confirmed by the approximating curves in the insets in Figure S3. The coefficient $a_{3}$ is related to the coefficient α of thermal expansion of the polymer matrix. The results of the approximation give overestimated values, which, apparently, can be associated with the simplified nature of the model used. At the same time, this simple reasoning gives a good qualitative explanation of the observed thermal behavior of the dielectric constant of MAE composites. 

### 3.3. Dependence of the MAE Capacitance on the Magnetic Field 

In this section, we examine the capacitance of the capacitor filled with MAE and placed in the magnetic field oriented perpendicular to the capacitor plates (see Figure 1). In contrast to works [22,23,41], in which capacitance measurements were carried out at a fixed distance between the capacitor plates, no external mechanical force was applied in this study, and the MAE sample could deform freely in the magnetic field. The magnetostriction phenomenon is well-known for MAE (see, for instance, [15,18,72,73]). 

**Figure 7:**
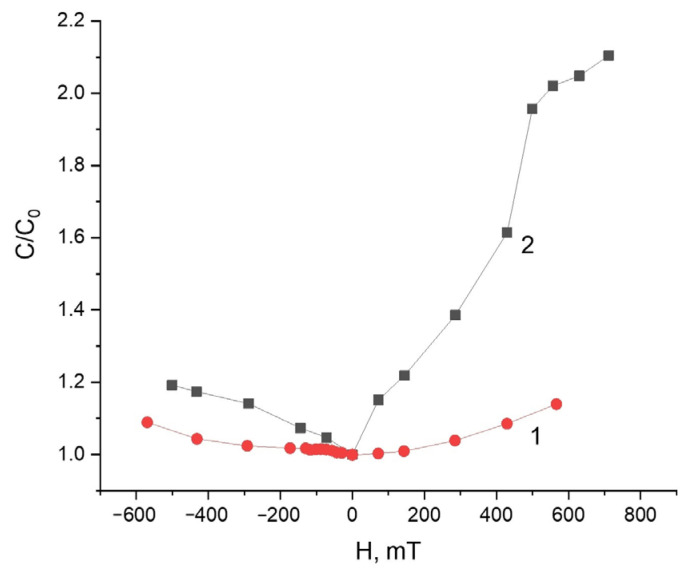
Magnetic field dependence of the relative capacitance of the capacitor filled with (**1**) an initial MAE–0 and (**2**) magnetized MAE–0–m, *f* = 1 MHz.

As seen from Figure 7, for a non-magnetized composite, the capacitance changes insignificantly in a magnetic field and this change is almost symmetric with respect to the direction of the field, while for a magnetized composite, the effect is much more pronounced and is distinguished by a considerable asymmetry. In the field opposite to the sample magnetization, the effect has the same magnitude as for the non-magnetized sample, while in the direction coinciding with the direction of the sample magnetization, the effect is several times greater. The effect can be partially explained by a change in the linear dimensions of the sample, as well as by some restructuring of the magnetic filler in an external field previously reported in Refs. [22,23]. A similar asymmetric response was observed in the viscoelastic behavior of magnetized MH MAEs in external magnetic fields [30].

## 4. Conclusions

In this work, the dielectric spectroscopy studies of MAEs containing magnetically hard NdFeB microparticles were performed in a wide temperature range. Two types of MAE samples were examined, which contained the same fraction of magnetic filler but differed by the filler composition. In the first series, a mixture of NdFeB and CI particles was used, while the samples of the second series were based on pure NdFeB particles. 

We showed that magnetization of the MAEs in a homogeneous magnetic field changes considerably their dielectric response. In particular, an increase in both relative permittivity and conductivity of MAEs was observed in the whole frequency range. For pure NdFeB composite at room temperature, 1.48-times increase in permittivity and 10-times increase in conductivity were reached at the frequency of 10 Hz. Even more, pronounced changes were observed for MAEs based on the mixture of NdFeB and CI particles. In particular, the value of $\varepsilon^{\prime }$ increased by 2.4 times, while the value of σ grew by almost two orders of magnitude at the same measurement conditions. The larger magnetic response of hybrid MAEs is presumably due to several reasons. These are MH particles that provide remanent magnetization of the sample after exposure to an external magnetic field, while MS iron particles are retained in clusters after removal of the field, forming chain-like structures in the field of MH particles. An order of magnitude mismatch in the particle size leads to their tighter packing within magnetic aggregates formed upon magnetization when smaller MS Fe particles can create additional bridges between larger MH particles which increases conductivity. Furthermore, it has recently been shown [39] that the presence of MS particles around larger MH particles causes an enlargement of magnetic moments, which the MH particles acquire during magnetization, and enhances the magnetic susceptibility and remanent magnetization of MAEs. Elucidating the influence of the NdFeB/Fe ratio on the magnetoelectric response of the composites is an interesting and important problem that is currently under study.

Comparative studies of the dielectric response of the original and magnetized samples allowed us to elucidate the role of the anisotropic distribution of magnetic filler induced by external magnetic fields. It was shown that in spite of a common mechanism of conductivity realized via hopping transport of carriers within both original and magnetized composites, a significant component of DC conductivity appears in the magnetized MAEs presumably due to a decreased distance between conducting metal particles in the course of their alignment along the lines of the applied magnetic field. Magnetization converts the composite from non-percolating to percolating state, apparently due to filler particle clusters becoming significantly anisotropic.

In addition, it was found that the hybrid MAEs exhibit a significant dependence of the electrical properties on the presence of a constant bias voltage and a change of the capacitance under the action of a homogeneous field which is asymmetric and depends on the mutual orientation of the sample magnetization and external magnetic field. More than a 2-times increase in the capacitance was observed in the field of 500 mT when the orientations of the applied field and magnetization field coincided. 

In general, we demonstrated that the dielectric and electric properties of hybrid MAEs could be successfully controlled by external magnetic fields. The obtained results are important for a fundamental understanding of their magneto-dielectric behavior as well as for practical applications of the developed materials, in particular, as sensors of the magnetic field. Furthermore, these materials can be considered as tunable dielectrics, which find numerous applications passive microwave components in phased array antenna, tunable filters, phase shifters, varactors and varicaps.

## Figures and Tables

**Table 1:** The composition of MAE samples.

Samples	Magnetic Filler Content, vol%	NdFeB/Fe Proportion	Magnetization State
MAE–Fe	35	80:20	-
MAE–Fe–m	35	80:20	magnetized
MAE–0	35	100:0	-
MAE–0–m	35	100:0	magnetized

## Data Availability

Data is contained within the article and Supplementary Materials.

## Supplementary Materials

The following are available online at https://www.mdpi.com/article/10.3390/polym13122002/s1, Figure S1: Size distribution of NdFeB particles. Figure S2: Temperature dependences of the dielectric constant, conductivity, the losses and the modulus of MAE–Fe and MAE–Fe–m. Figure S3: Temperature dependences of the dielectric constant and conductivity of MAE–0 and MAE–0–m.
