# Supplementary material for: Dielectric Spectroscopy of Hybrid Magnetoactive Elastomers
Source: Polymers (Basel). 2021 Jun 18;13(12):2002. doi: 10.3390/polym13122002 (PMC8235524; doi:10.3390/polym13122002)
Supplement: Supplementary file 1 [file polymers-13-02002-s001.zip › polymers-1250605-supplementary.pdf]

Supplementary Materials

Dielectric Spectroscopy of Hybrid Magnetoactive Elastomers

Vitaliy G. Shevchenko [1], Gennady V. Stepanov [2,3] and Elena Yu. Kramarenko [3,4,*]

[1] Enikilopov Institute of Synthetic Polymeric Materials of Russian Academy of Sciences (ISPM RAS), 117393 Moscow, Russia; vgshev@gmail.com
[2] State Scientific Center of the Russian Federation, Institute of Chemistry and Technology of Organoelement Compounds, 111123 Moscow, Russia; gstepanov@mail.ru
[3] Faculty of Physics, Lomonosov Moscow State University, 119991 Moscow, Russia; kram@polly.phys.msu.ru
[4] A. N. Nesmeyanov Institute of Organoelement Compounds RAS, 119991 Moscow, Russia
* Correspondence: kram@polly.phys.msu.ru; Tel.: +7-495-939-4013

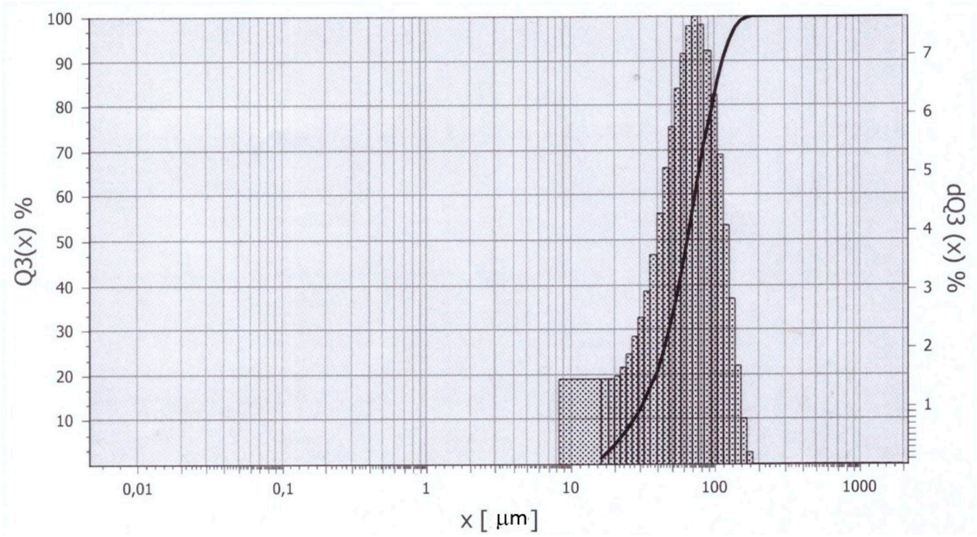

Figure S1. Size distribution of NdFeB particles (Analysette 22, Fritsch). Before measurements, the particles passed through a sieve with a meshsize of 100 µm. The shift in the distribution to larger sizes is caused by the irregular shape of the particles and their improper handling of the device.

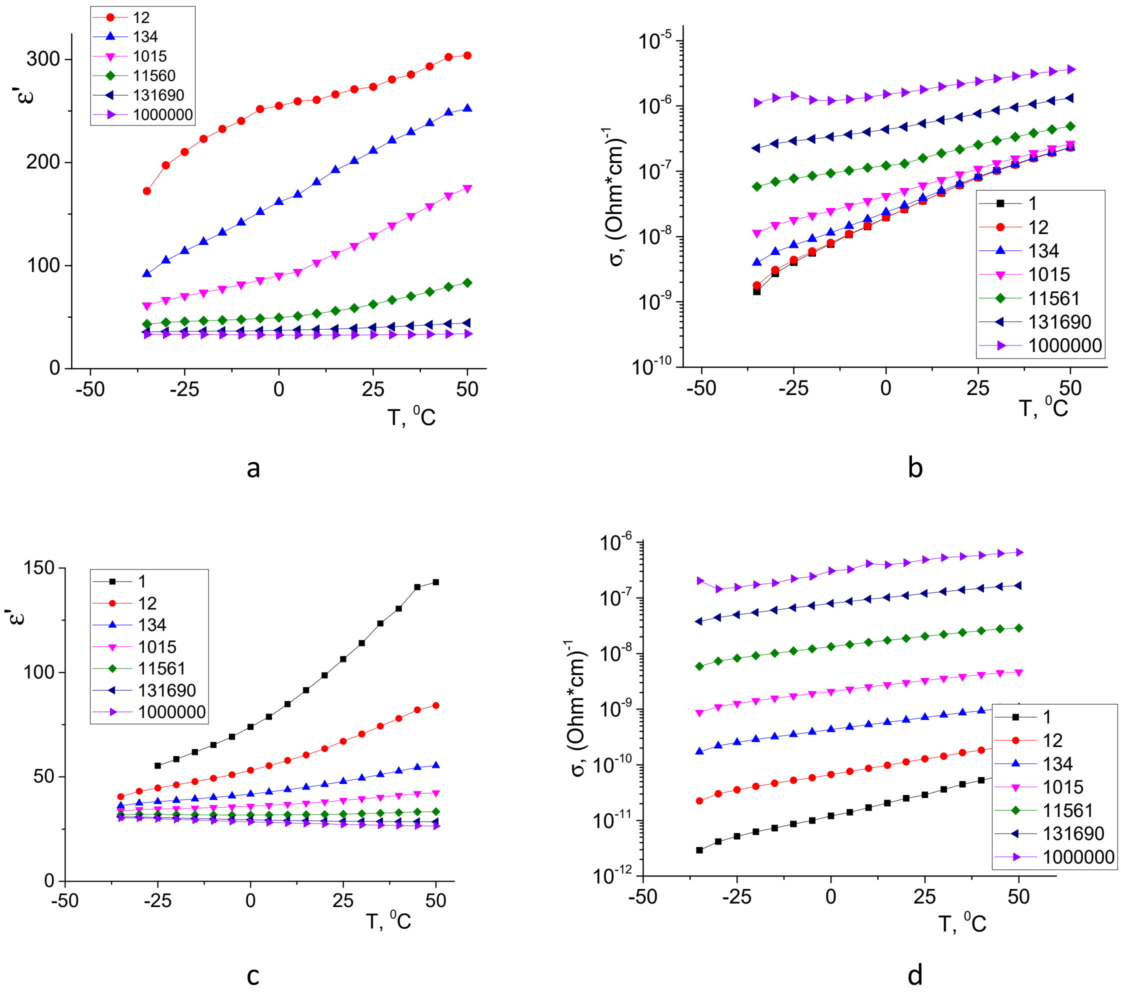

Figure S2. Temperature dependence of the dielectric permittivity (a,c) and conductivity (b,d) of the initial MAE-Fe (c,d) and magnetized MAE-Fe-m (a,b) samples at various frequencies as indicated in Hz.

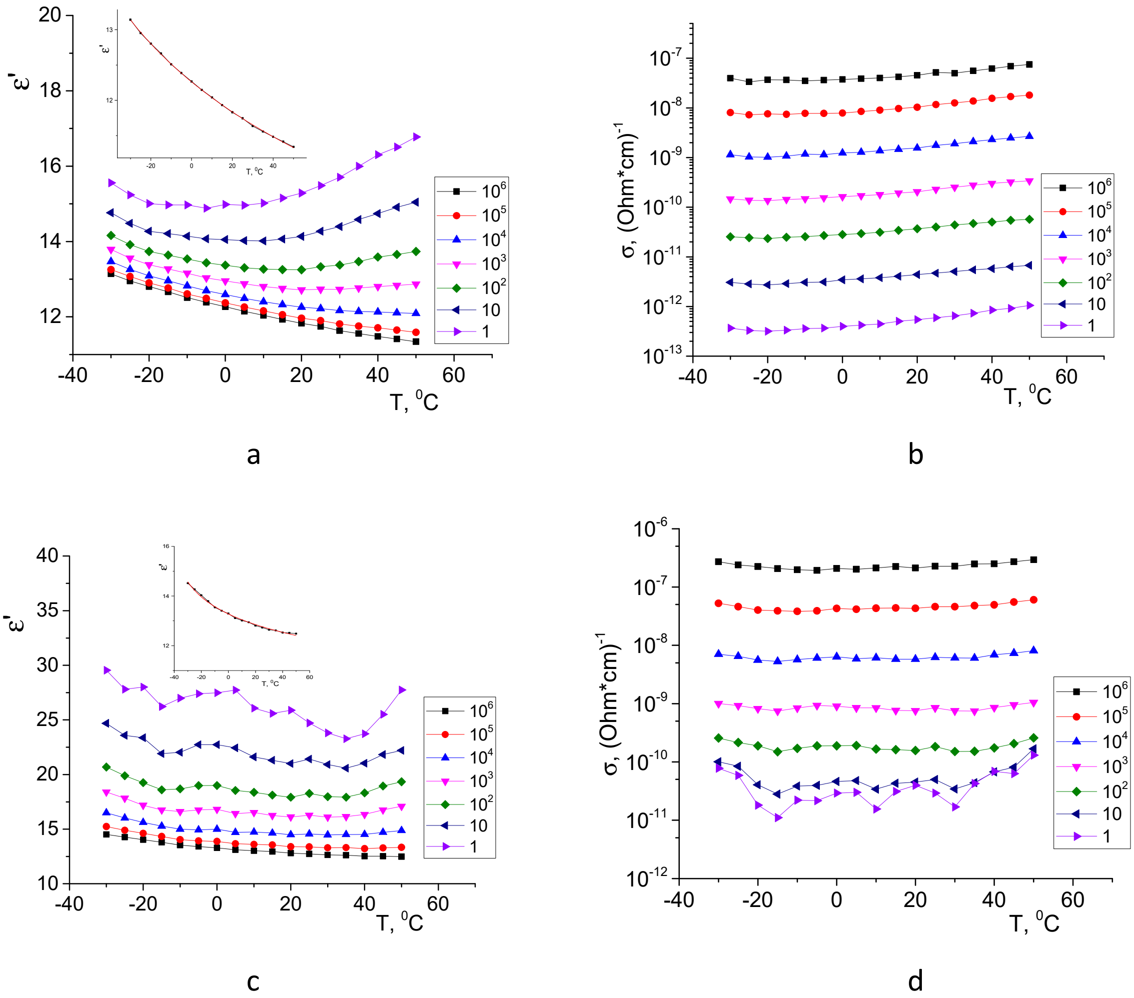

Figure S3. Temperature dependence of the dielectric permittivity (a,c) and conductivity (b,d) of the initial MAE-0 (c,d) and magnetized MAE-0-m (a,b) samples at various frequencies as indicated in Hz.
